# Inter-observer agreement using the LI-RADS version 2018 CT treatment response algorithm in patients with hepatocellular carcinoma treated with conventional transarterial chemoembolization

**DOI:** 10.1007/s00261-021-03272-9

**Published:** 2021-09-28

**Authors:** Krzysztof Bartnik, Joanna Podgórska, Grzegorz Rosiak, Krzysztof Korzeniowski, Olgierd Rowiński

**Affiliations:** 1 Doctoral School, Medical University of Warsaw, Warsaw, Poland; 2 Second Department of Radiology, Medical University of Warsaw, Ul. Banacha 1a, 02-097 Warsaw, Poland

**Keywords:** TACE, Hepatocellular carcinoma, LI-RADS treatment response, CT

## Abstract

**Aim:**

To determine inter-reader agreement in categorization of imaging features using the Liver Imaging Reporting and Data System (LI-RADS) treatment response (LR-TR) algorithm in patients with hepatocellular carcinoma (HCC) treated with conventional transarterial chemoembolization (cTACE).

**Methods:**

Two radiologists used the LR-TR algorithm to assess 112 computed tomography (CT) examinations of 102 patients treated with cTACE. The inter-observer agreement in categorization of LR-TR features was assessed using kappa (κ) statistics.

**Results:**

There was substantial inter-observer agreement between the two reviewers using the LR-TR algorithm (κ = 0.70; 95% CI 0.58–0.81). The two reviewers categorized tumors as non-viable in 37 (33.0%) and 39 (34.8%) of 112 examinations, viable in 58 (51.8%) and 62 (55.4%) examinations, and equivocal in 18 (16.1%) and 11 (9.8%) examinations, respectively. There was almost perfect inter-observer agreement for the LR-TR non-viable category (κ = 0.80; 95% CI 0.68–0.92), substantial agreement for the viable category (κ = 0.78 95% CI 0.67–0.90), and fair agreement for the equivocal category (κ = 0.25; 95% CI 0.02–0.49).

**Conclusion:**

The LR-TR algorithm conveys high degrees of inter-observer agreement for the assessment of CT imaging features in the viable and non-viable categories. Further refinement of indeterminate features may be necessary to improve the correct categorization of equivocal lesions.

**Graphic abstract:**

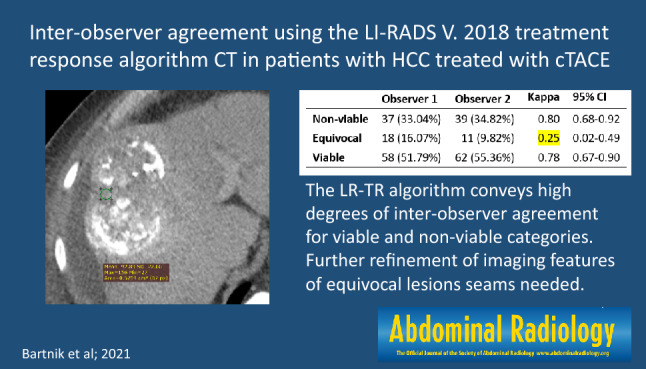

## Introduction

Locoregional treatments, including conventional transarterial chemoembolization (cTACE), play an important role in the treatment of patients with unresectable hepatocellular carcinoma (HCC) [1, 2]. Accurate assessment of treatment response and HCC viability is an essential part of patient management [3]. Treatment response can be evaluated using contrast-enhanced imaging together with various currently available response algorithms [4, 5]. Several systems have been developed to date with the aim of standardizing the evaluation of treatment response, including the Liver Imaging Reporting and Data System (LI-RADS) treatment response (LR-TR) algorithm [6, 7]. This new HCC-specific response algorithm was introduced in 2018 and is designed to assess treatment response following locoregional therapies using computed tomography (CT) or magnetic resonance imaging (MRI) [8, 9]. The LR-TR algorithm aims to improve communication between health-care providers, facilitate patient care, and standardize evaluation of treatment response for clinical and research purposes.

Arterial phase hyperenhancement (APHE) is consistently emphasized as the most important post-treatment feature associated with residual tumor viability and is adopted in many treatment response systems such as the modified Response Evaluation Criteria in Solid Tumors (mRECIST) and World Health Organization (WHO) criteria [10–13]. One key distinctive feature of the LR-TR algorithm is the inclusion of additional imaging features of tumor viability, such as washout and enhancement similar to pretreatment [14]. Although the LR-TR algorithm has significantly improved the evaluation of treatment response following locoregional therapies in HCC, its true reliability for surveillance after cTACE remains unclear. Previous studies showed varying degrees of inter-reader agreement using the LR-TR criteria, with conclusions limited by substantial heterogeneity in imaging methods and the locoregional treatments analyzed [15, 16].

To our knowledge, no single study to date has specifically investigated categorization of CT examinations using the LR-TR algorithm in patients treated with cTACE. Given the increasing frequency of use of the LR-TR algorithm in clinical practice, this study aimed to assess the performance and repeatability of the LR-TR algorithm for post-TACE evaluation of treatment response in treatment-naive patients with HCC. Inter-observer agreement in categorization of CT imaging features and assessment of LR-TR categories was further explored.

## Materials and methods

In this retrospective study we analyzed data for consecutive patients with HCC who underwent repeated cTACE procedures as initial therapy between March 2016 and January 2018. The study was approved by the local Institutional Ethical Committee of Human Experimentation and complied with the current version of the Declaration of Helsinki.

We included patients who (1) had unresectable HCC who underwent cTACE therapy, (2) had at least one HCC observation confirmed by dynamic contrast-enhanced CT imaging (American Association for the Study of Liver Diseases criteria, OPTN classes 5A, 5B and 5X); (3) had no HCC-specific therapy before enrollment; (4) had an available post-treatment dynamic contrast-enhanced CT liver examination within 90 days following cTACE; and (5) had complete clinical data. The exclusion criteria were as follows: (1) image omission, degradation, or incompatibility with the LI-RADS 2018 technical recommendations; (2) other HCC-specific therapy between cTACE and treatment outcome evaluation (e.g., combined TACE-ablation).

### Image analysis

All patients underwent post-treatment contrast-enhanced CT and two independent abdominal radiologists (with 5 and 8 years of experience in liver CT, respectively) reviewed each patient’s post-treatment CT examination using the LR-TR criteria. Both readers were blinded to clinical information, but were aware that patients had undergone cTACE for HCC.

On post-treatment CT scans, each reader assessed the presence of the following imaging features: (1) nodular, mass-like, or irregular thick tissue in or along the treated lesion with APHE, (2) expected treatment-specific enhancement, and (3) washout.

Moreover, the following variables were also included in the analysis: presence of indeterminate enhancement pattern and treatment response category (non-viable, equivocal, or viable). The criteria for LR-TR categories are listed in Table 1. If an observation was no longer visible after treatment, the lesion was categorized as LR-TR non-viable.

**Table 1:** The criteria for LR-TR categories

Response category	Criteria
LR-TR non-viable	No lesional enhancement or treatment-specific expected enhancement pattern
LR-TR equivocal	Atypical enhancement pattern, not meeting criteria for non-viable or viable category
LR-TR viable	Presence of any of the following: APHE or washout appearance or enhancement similar to pretreatment

**Fig. 1:**
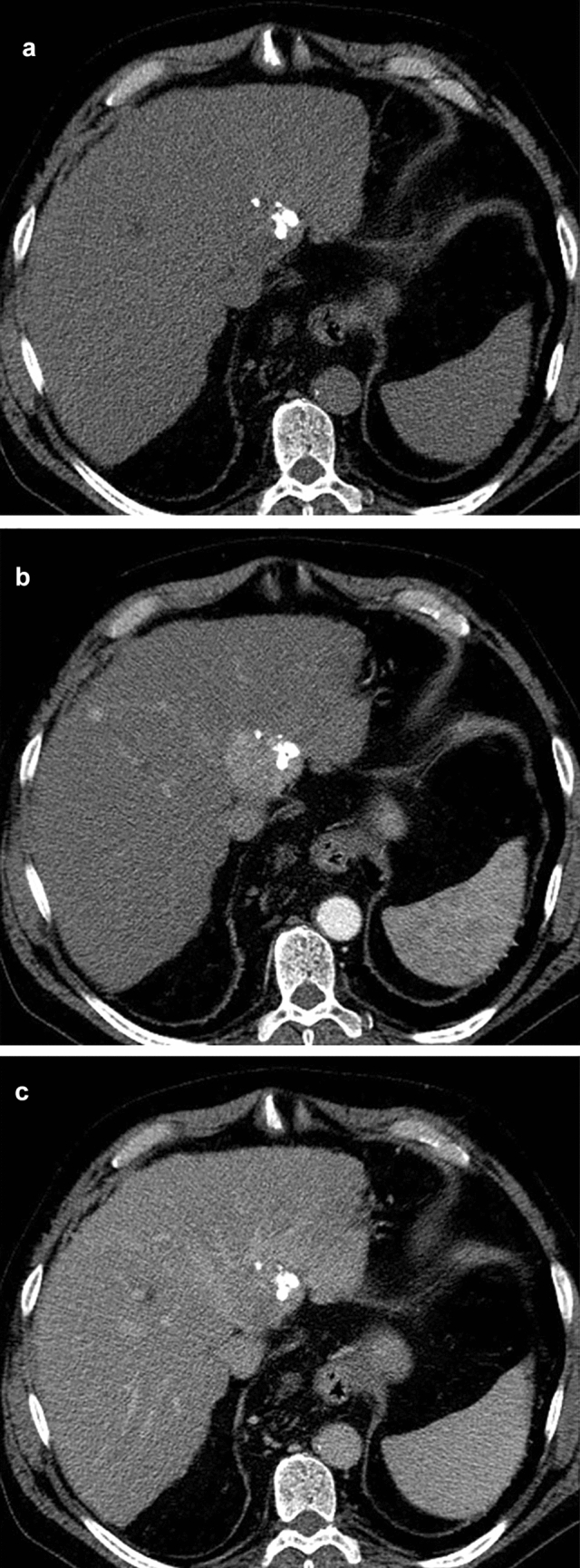
Multiphase CE-CT shows a treated lesion only partially filled with lipiodol. In major part of the lesion mass-like arterial phase hyperenhancement is seen followed by washout appearance in the portal venous phase suggesting post-treatment tumor viability

**Fig. 2:**
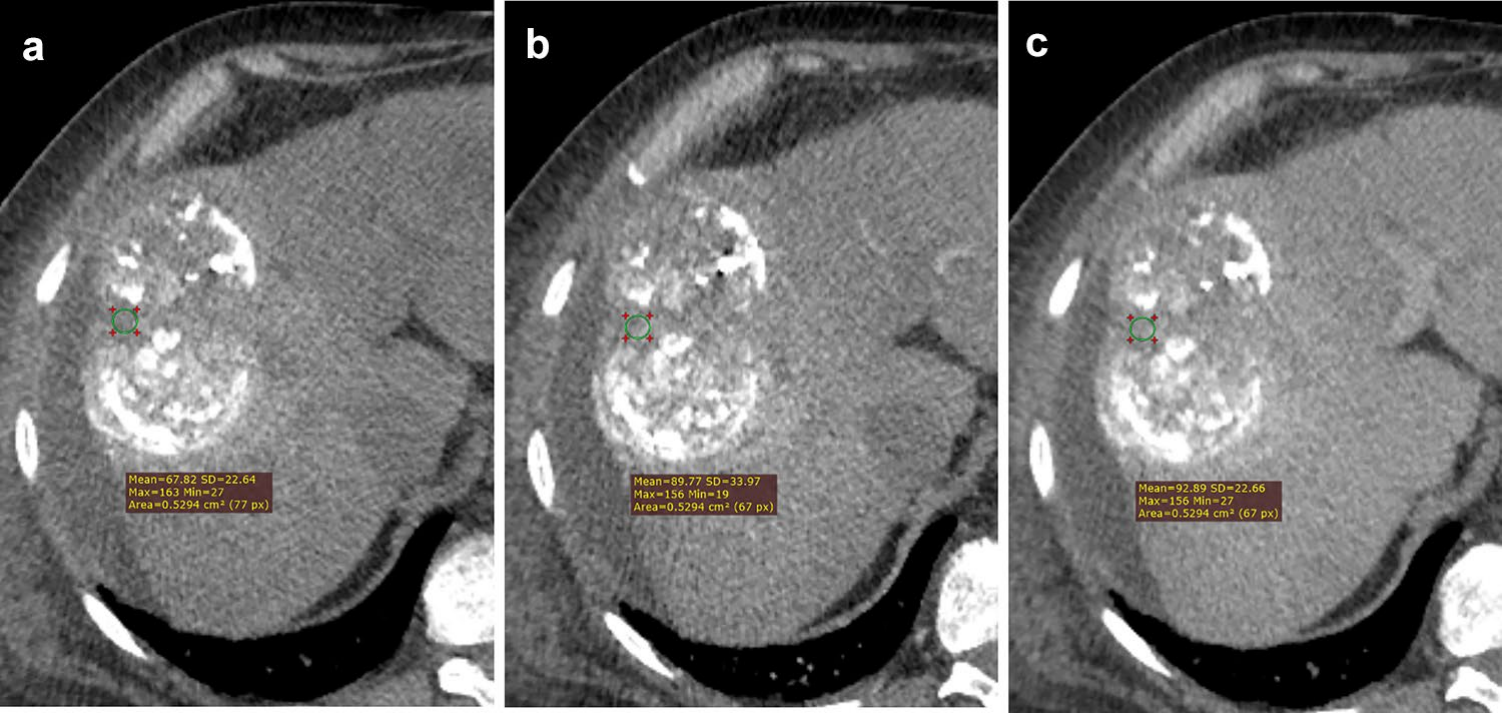
A large, treated lesion is seen with scattered, inhomogeneous deposition of lipiodol, arterial phase hyperenhancement, or washout appearance are not definite, but in few tumor regions tissue enhancement is detected and therefore LR-TR equivocal category is most appropriate

**Fig. 3:**
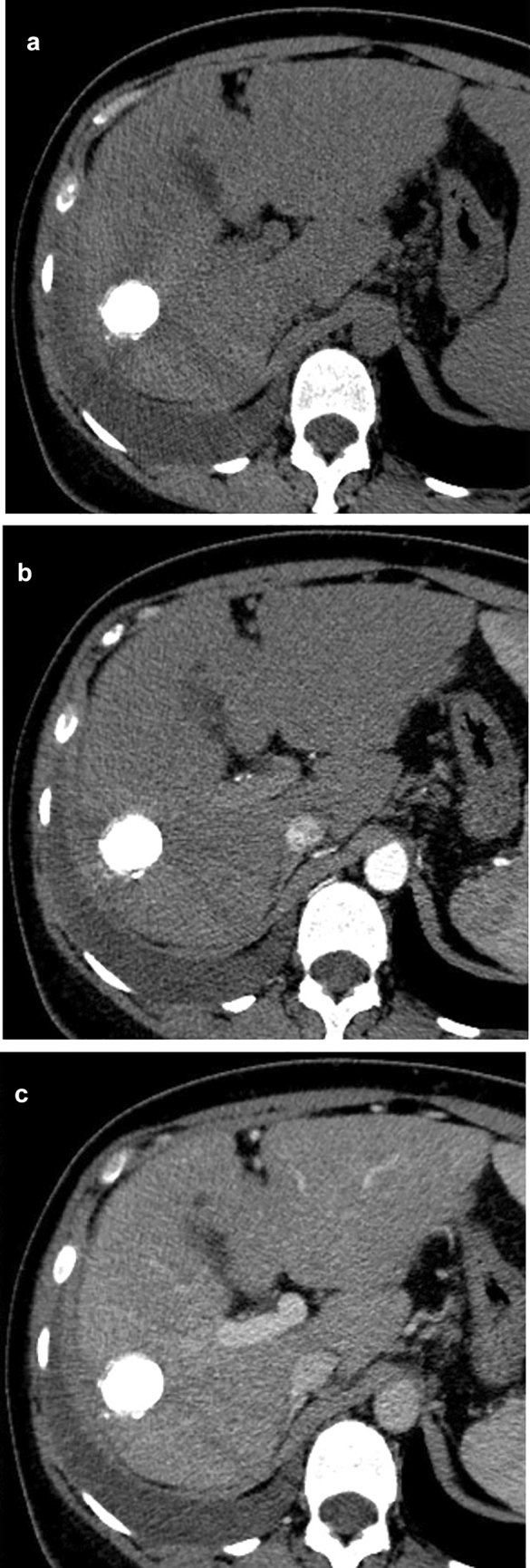
The treated lesion is completely filled with lipiodol, with no lesional enhancement, meeting the criteria for LR-TR non-viable

If the patient had more than one treated observation, each representing different treatment responses, the observation reflecting the least favorable response was chosen and reported by its segmental location to ensure that each observer assessed the same lesion. Consequently, only one treated observation per patient was included in the statistical analysis. On that basis, the final response category was reported in aggregate, in line with the LI-RADS guidelines. Examples of observations where there was a consensus of both observers are shown in Figs. 1, 2, and 3. In cases of radiologist uncertainty between two LR-TR categories, a tie-breaking rule was applied to choose the category reflecting the lower certainty, according to the LI-RADS guidelines [17, 18].

### TACE technique

All patients underwent a standard cTACE procedure. After obtaining femoral access, hepatic and tumoral blood supply were examined. Vessels feeding target observations were selectively catheterized using a microcatheter and 20–40 mL of a mixture of lipiodol and doxorubicin in a 1:1 ratio was subsequently slowly injected until arterial flow stasis was observed. Then, embolization with gelatin sponge particles (Spongostan absorbable haemostatic gelatin sponge, Ethicon Inc) was performed. The TACE session was repeated after 4–6 weeks when indicated and feasible. A standard embolization cycle consisted of two (or three, if indicated) TACE sessions and subsequent CT examination.

### Statistics

SAS software (Statistical Analysis System version 9.4, SAS Institute Inc., Cary, NC, USA) was used to perform the statistical analyses. Categorical variables are reported as counts and percentages. The kappa coefficient (κ) was used to assess inter-observer agreement for each of the LR-TR features. The standard kappa values were defined as: < 0: no agreement; 0–0.20: slight; 0.21–0.40: fair; 0.41–0.60: moderate; 0.61–0.80: substantial; and 0.81–1.0: perfect.

## Results

### Patient population

In total, 102 patients were enrolled, with a median age of 65 years at the time of treatment initiation. Patients’ baseline characteristics are summarized in Table 2. Overall, 61 (60%) patients at baseline had a single treated observation and 41 (40%) had two or more lesions. Fifty-four (54%) patients had intermediate-stage (B) HCC according to Barcelona Clinic Liver Cancer (BCLC) staging. Unequivocal tumor invasion into the portal vein was noted in nine during the follow-up period. Viral hepatitis was the most common cause of liver disease (70 [69%] of 102 patients), and 86 (84%) patients had preserved liver function (Child–Pugh–Turcotte class A). Overall, 112 CT studies were analyzed by each observer. Inter-observer agreement for LR-TR categories and imaging features is shown in Table 3.

**Table 2:** Demographic and clinical characteristics of patients

Baseline characteristic	No. of patients	(%) or (range)
Age (years)		
Median	65 (58–72)	(43–88)
< 60	30	29.7
> 60	71	70.3
Gender		
Male	77	75.5
Female	25	24.5
Chronic liver disease etiology		
HBV	12	11.7
HCV	46	45.1
Alcoholic	27	26.5
Mixed	2	2.0
Other	15	14.7
CPT class		
A	86	84.3
B	16	15.7
BCLC stage		
A	48	47.1
B	54	53.9
Serum AFP		
< 200 ng/mL	69	67.7
≥ 200 ng/mL	33	32.4
ALBI		
1	57	55.9
2	40	39.2
3	5	4.9
Number of treated observations		
1	61	59.8
2	23	22.6
≥ 3	18	17.7

*HBV* Hepatitis B virus, *HCV* Hepatitis C virus, *CPT* Child–Pugh–Turcotte, *BCLC* Barcelona clinic liver cancer, *AFP* α-fetoprotein, *ALBI* albumin–bilirubin grade

**Table 3:** Inter-observer agreement for LR-TR categories and LI-RADS TR imaging features

	Observer 1 (%)	Observer 2 (%)	Kappa	95% CI
Overall	112 (100)	112 (100)	0.70	0.58–0.81
Non-viable	37 (33.04)	39 (34.82)	0.80	0.68–0.92
Equivocal	18 (16.07)	11 (9.82)	0.25	0.02–0.49
Viable	58 (51.79)	62 (55.36)	0.78	0.67–0.90
APHE	57 (50.89)	61 (54.46)	0.79	0.67–0.90
Washout	47 (41.96)	52 (46.43)	0.69	0.56–0.83
Expected post-treatment enhancement	19 (16.96)	5 (4.46)	0.19	− 0.03–0.41
Indeterminate enhancement	24 (21.43)	9 (8.04)	0.35	0.13–0.56

*APHE* arterial phase hyperenhancement

### Inter-observer agreement for LR-TR categories

There was substantial inter-observer agreement between the two reviewers for per-session treatment response according to the LR-TR algorithm (κ = 0.70; 95% CI 0.58–0.81). Tumors in 37 (33.0%) and 39 (34.8%) of 112 examinations were classified as non-viable, 58 (51.8%) and 62 (55.4%) as viable, and 18 (16.1%) and 11 (9.8%) as equivocal by the two reviewers, respectively. There was almost perfect inter-observer agreement for the LR-TR non-viable category (κ = 0.80; 95% CI 0.68–0.92), substantial agreement for the LR-TR viable category (κ = 0.78 95% CI 0.67–0.90), and only fair inter-observer agreement for the LR-TR equivocal category (κ = 0.25; 95% CI 0.02–0.49). A substantial proportion of patients with lesions categorized as LR-TR equivocal by the two observers [10 (56%) of 18 and 5 (45%) of 11, respectively] were considered not to have achieved local tumor control by the institutional tumor board and were referred for additional TACE sessions.

### Inter-observer agreement for LR-TR features

For post-treatment CT features, the highest agreement was observed for the presence of APHE (κ = 0.79; 95% CI 0.67–0.90). The washout feature had substantial inter-reader agreement, with κ = 0.69 (95% CI 0.56–0.83). Of note, the washout feature was observed by one of the radiologists independently of APHE in 2 (2%) patients. Agreement between readers was lowest for the expected post-treatment enhancement (κ = 0.19; 95% CI − 0.03 to 0.41) and indeterminate hyperenhancement (κ = 0.35; 95% CI − 0.05 to 0.60) features.

## Discussion

A large and growing body of literature has investigated the reliability of the LR-TR algorithm for lesion classification in CT and MRI examinations in patients with HCC [19–23]. A recent meta-analysis by Kim et al. showed substantial overall inter-reader agreement when the LR-TR algorithm was used to assess treatment response following locoregional therapies [15]. Nevertheless, the studies included in that meta-analysis suffered from substantial study group heterogeneity, which was significantly associated with differences in the study designs and treatment modalities used, indicating that more research is required on this topic. The current study addresses this gap by assessing inter-reader agreement in the categorization of LR-TR features on CT-only examinations in a homogeneous group of patients undergoing cTACE. The most striking result of our analysis is that the features that comprise the equivocal category had unsatisfactorily low reproducibility, with indeterminate hyperenhancement being the dominant post-treatment imaging feature. Consequently, low inter-reader agreement was observed for the equivocal treatment response category. This is in line with the results of a recent study by Shropshire et al., in which post-TACE CT or MRI examinations of a group of 45 adult patients were analyzed [24]. The authors showed low inter-reader agreement for the indeterminate hyperenhancement feature (κ = 0.25) as well as moderate agreement for the final LR-TR assessment category (κ = 0.55). Inter-reader agreement is essential for standardized reporting systems, such as LI-RADS and the LR-TR algorithm. An implication of these findings is that the next update of the LR-TR guidelines should focus on simplifying or clarifying ambiguous criteria in order to improve inter-reader agreement.

Of note, a substantial number of patients classed as having an equivocal treatment response in our study were considered not to have achieved local disease control by the tumor board and were referred for subsequent TACE sessions. This also highlights the need for discussion of patients with equivocal tumor responses in a multidisciplinary tumor board setting. Importantly, previous analysis showed no significant differences in overall survival between patients with an initial equivocal response and those with a viable response [25]. This correlates with the results of previous studies, in which most of the equivocal observations were incompletely necrotic at histopathologic examination [21, 24]. Taken together, these results suggest that more attention is needed for this group of patients. Currently, the LI-RADS guidelines advocate 3-month imaging intervals for both equivocal and non-viable tumors. The results of this study support the idea that LR-TR equivocal lesions may require closer imaging follow-up and possibly further treatment as many patients might benefit from additional locoregional therapies. Future studies will benefit from elucidating whether more aggressive management of patients with an equivocal treatment response could help to improve outcomes following locoregional treatment.

Overall, the inter-observer agreement was highest for the APHE feature, followed by the washout appearance, with both features showing high κ values, consistent with previous studies using the LR-TR algorithm [26, 27]. Recent studies with radiologic–pathologic correlation have confirmed that APHE is the most reliable marker of incomplete tumor necrosis and is the post-embolization feature with the greatest inter-reader agreement [21, 24]. One key difference between the LR-TR algorithm and other systems is the inclusion of the washout feature as an additional marker of tumor viability. Shropshire et al. noted that none of the post-embolization examinations showed the washout feature independently of APHE [24]. In our study, washout was observed independently of APHE in a small subset of patients, facilitating classification of viable responses. This finding supports the incorporation of additional imaging features into the LR-TR categorization in order to improve performance compared with the APHE feature alone. Of note, inter-reader association for “enhancement similar to pretreatment” feature was not calculated because of a small sample size (only five and four of such features recorded by the observers, respectively). A similar observation was reported in the study by Shropshire et al. where this trait was also not included in the analysis for same reason. In the current analysis contrast-enhanced post-treatment CT was used in all study participants. This could be viewed as a strength of this study given that the inclusion of different diagnostic imaging modalities may interfere with the assessment of treatment response. It is worth noting that assessment of treatment response on CT and MRI is treated equally in the LI-RADS guidelines, but the imaging method may significantly affect the quality of assessment following locoregional HCC therapies. For example, iodized oil accumulation on CT evaluation following cTACE portends a satisfactory result, but also makes identifying areas of viable tumor tissue difficult [28]. By contrast, iodized oil does not interfere with the detection of residual tumor using MRI [27]. Although MRI could potentially overcome the effect of iodized oil, CT is still used in many institutions partly because it is more accessible. Nevertheless, no studies have so far directly compared the efficacy of CT and MRI in assessing responses following locoregional therapies in patients with HCC. It would be interesting to compare patient outcomes in groups assessed separately using these two imaging methods.

## Limitations

Finally, a number of important limitations need to be considered. First, our study population consisted solely of patients treated with cTACE. This reduced heterogeneity but limited the generalizability of our results to patients treated with other locoregional therapies, such as ablation or radioembolization. Second, a per-session approach was adopted to assess post-treatment responses with the aim of simplifying clinically relevant findings and communicating impressions as clearly as possible. Such an approach is frequently used in clinical practice to facilitate communication among the tumor board, where not only radiologists but also surgeons, clinical oncologists, and pathologists take part in patient management. This means that the final category of response has been reported in aggregate to determine whether or not a specific patient requires further locoregional treatment. In clinical practice, whether and how to treat individual patients depends largely on overall local tumor control [29]. A per-session approach could adversely affect inter-observer agreement; however, in this study, one dominant observation was highlighted in the case of multiple tumor loci for a single patient. These observations were tagged by observers to minimize the risk of potential bias. Importantly, the LR-TR algorithm enables per-lesion categorization of treated observations based on the post-treatment imaging features of treated tumors. However, if a patient has more than one target lesion, the LI-RADS guidelines allow us to choose whether to report treated observations separately, in aggregate, or as a combination of both to communicate the results as clearly as possible [18].

## Conclusion

The LR-TR algorithm conveys high degrees of inter-observer agreement for the categorization of APHE and washout features of treated observations after cTACE. Consequently, the assessment of viable and non-viable categories is highly repeatable, but further refinement of indeterminate features may be necessary to improve the correct categorization of imaging features for patients with an equivocal lesion. Future studies may benefit from simplifying or clarifying ambiguous criteria in order to improve inter-reader agreement.

## Data Availability

Data available.
